# An error-aware gaze-based keyboard by means of a hybrid BCI system

**DOI:** 10.1038/s41598-018-31425-2

**Published:** 2018-09-04

**Authors:** Fotis P. Kalaganis, Elisavet Chatzilari, Spiros Nikolopoulos, Ioannis Kompatsiaris, Nikos A. Laskaris

**Affiliations:** 1 Aristotle University of Thessaloniki, Department of Informatics, AIIA lab, Thessaloniki, 54124 Greece; 2 Centre for Research and Technology Hellas, Information Technologies Institute, MKlab, Thessaloniki, 57001 Greece; 3 Aristotle University of Thessaloniki, Neuroinformatics Group, Thessaloniki, 54124 Greece

## Abstract

Gaze-based keyboards offer a flexible way for human-computer interaction in both disabled and able-bodied people. Besides their convenience, they still lead to error-prone human-computer interaction. Eye tracking devices may misinterpret user’s gaze resulting in typesetting errors, especially when operated in fast mode. As a potential remedy, we present a novel error detection system that aggregates the decision from two distinct subsystems, each one dealing with disparate data streams. The first subsystem operates on gaze-related measurements and exploits the eye-transition pattern to flag a typo. The second, is a brain-computer interface that utilizes a neural response, known as Error-Related Potentials (ErrPs), which is inherently generated whenever the subject observes an erroneous action. Based on the experimental data gathered from 10 participants under a spontaneous typesetting scenario, we first demonstrate that ErrP-based Brain Computer Interfaces can be indeed useful in the context of gaze-based typesetting, despite the putative contamination of EEG activity from the eye-movement artefact. Then, we show that the performance of this subsystem can be further improved by considering also the error detection from the gaze-related subsystem. Finally, the proposed bimodal error detection system is shown to significantly reduce the typesetting time in a gaze-based keyboard.

## Introduction

Brain-Computer Interfaces (BCIs) have been widely employed for providing alternative communication and control options to both disabled and able-bodied people. Several relevant applications were emerged, including information recommender systems^1^, spellers^2,3^, robotic devices^4^ and wheelchair controllers^5^. Supported by advances in machine learning, and in conjunction with the ever-increasing availability of consumer EEG scanners, BCIs are currently incorporated into multi-modal systems as well, leading to improved, adaptable, versatile and natural interfaces.

A hybrid, also referred to as multi-modal, BCI (hBCI or mmBCI) establishes a pathway between the user and the computer based on distinct types of brain activity or by combining brain signals with other physiological signals, such as eye gaze, electrocardiography and electromyography^6^. The hBCIs are considered as capable of alleviating the restrictions of BCIs associated with the detection of patterns in noisy neural data and, hence, they are more suitable for non-clinical applications.

Over the last years, research in BCIs has managed to achieve significant improvement in terms of detecting the users’ intentions^7^. However, in a real-world setting, the interpretation of brain commands still remains an error-prone procedure leading to inaccurate interactions, probably due to the underlying oversimplification that the brain is occupied at most by the task we are trying to detect patterns for which constitutes a major barrier for the wider deployment of BCIs. Even for multi-modal interaction schemes, the attained performance is far from optimal. As a means to overcome these debilities, and apart from developing more sophisticated machine-learning techniques or adding further modalities, scientists have also exploited the users’ ability to perceive errors. The principal idea is that a BCI system may incorporate, as feedback, the user’s judgement about its function and use this feedback to correct its current output. Towards this direction, the most common approach is to detect Error-Related Potentials (ErrPs), a special type of Event-Related Potentials (ERPs) that appear shortly after the user recognizes an error. Previous studies have shown that ErrPs can be utilized to correct spelling errors in P300 spellers^8^, as well as to adapt the classifiers, reduce the calibration time and improve the performance of BCIs based on code-modulated Visual Evoked Potentials (c-VEP)^9^.

The vast majority of BCI systems employs electroencephalography (EEG), that is designed to record the electric potential signals produced by the synchronous action of neurons with parallel geometric orientation^10^. However, EEG also captures the electrical activity from sources other than the brain. It seems, therefore, difficult for EEG to be effectively combined with other modalities that involve ocular or muscular movement, such as the eye-gaze captured by an eye-tracking device. Despite the fact that ocular artefacts may contaminate the recorded brain activity, EEG and eye-tracking have been successfully combined during the last years. One such application concerns the identification and removal of artefacts in EEG signals using the eye-activity traces^11^ and the differentiation between intentional and spontaneous eye movements^12^. Another one, combines EEG and eye tracking to disentangle perceptual/attentional/cognitive factors affecting reading. Neural activity is associated with the displayed items by tracking the eye movement and isolating the neural signal around the gaze fixation onsets^13,14^.

In the presented work, we investigate whether EEG can be used in conjunction with an eye-tracker towards the development of a high-speed gaze-based keyboard. Our study proceeds in two distinct directions. Firstly, we demonstrate that a specific neurophysiological event associated with error perception is elicited just after the user realizes a typesetting error. An associated activation pattern, free from eye-related artefacts, can be robustly detected and used to flag the errors. Secondly, we provide evidence that this event, which is a special case of an ErrP, can serve as the basis for an automated Error Detection System (EDS) that is operated directly by the user’s brain responses and may be complemented by information extracted from the eye-movement patterns to further boost its performance. Apart from verifying the theoretical improvement in the typing speed of a gaze-based keyboard, we also provide experimental results from an on-line simulation, where the proposed approach is compared against a regular gaze-based typesetting scenario, without any error-detection assistance. To the best of our knowledge, this is the first study that attempts to combine gaze-based typesetting with ErrPs’ decoding and demonstrates that such an integration holds promise for an enhanced user experience.

## Methodology

**Figure 1:**
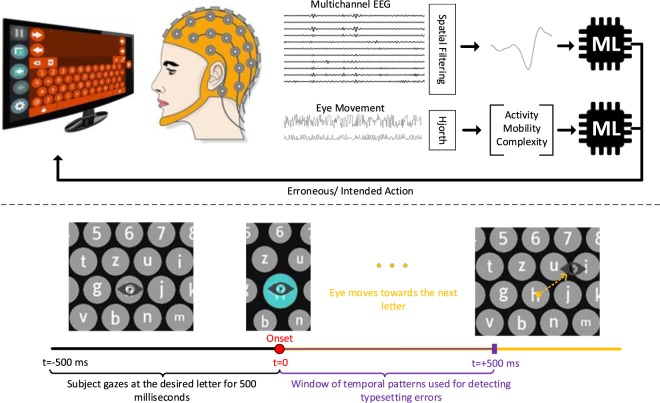
(Top) Schematic outline of the error-aware keyboard. A hybrid BCI system, relying on brain activity patterns and eye-movement features, detects and deletes characters that are mistyped. The depicted Machine Learning (ML) modules correspond to linear SVMs. (Bottom) Timeline describing the sequence of events during the typesetting experiment. Initially, the participant starts gazing at the desired letter. When he completes a 500 ms time-interval of continuous gazing, the key is registered and simultaneously the associated visual indication is presented. The physiological responses following this indication are used to detect typesetting errors. We note that the “eye” icon was not presented in the experiments and it is only shown here for presentation clarity purposes.

In our experiments the electrical brain activity and gazing location were continuously recorded while the subject was performing a gaze-based typing task (Fig. 1). The data acquisition protocol was designed so as to uncover the physiological patterns associated with the perception of an erroneous visual key press (i.e. key registration). The discovered patterns that stem from the participants brain and eye activity are then exploited by a machine learning scheme in order to realize a gaze-based keyboard with an automatic error-detection capability. An extended overview of the experimental setup can be found in the *Materials and Methods: Experimental Protocol*.

### Typing Task and Physiological recordings

The recording protocol relied on a standard gaze-based keyboard paradigm that was implemented by an eye-tracker attached to a pc monitor^15,16^. The gazing information, in the form of a densely sampled sequence of x-y coordinates corresponding to the eye trace on the screen, was registered simultaneously with the participant’s brainwaves. The purpose of this experiment was to provide data where patterns in the physiological activity, of either brain or/and eyes, could be associated with the case of a typo (due to either the inaccuracy of the eye-tracker or a human mistake). In the study of event-related neurophysiological responses, the precise timing is of paramount importance. For this reason, the functionality of the gaze-based typesetting system had to be modified. Typical gaze-based keyboards use a visual indication, shown in Supplementary Fig. 2, to continuously inform the user about the gaze location. A visual key is registered, only, after the user has constantly gazed at it for a certain amount of time, usually referred to as dwell time. However, this visual feedback notifies the user on the typing result at arbitrary times and as such the ErrPs are not time-locked to the registration of the visual key. This option of continuous visual feedback was deactivated in our experimental setup in order to ensure that transient brain responses, time-locked to erroneous typesetting, would be elicited. It was only after a stared key had been typed (or, equivalently, gazed at for more than 0.5 seconds) that appeared as selected (refer to Supplementary Fig. 2). In this way, the perception of a typo could be associated with a specific timestamp. In other words, the onset of a wrong selection was the trigger for an ErrP-response.

Twenty sentences, which can be found in Supplementary Table 1, were provided sequentially to the subjects with the instruction to type them with the adjusted gaze-based keyboard. The current sentence was not accessible to the subjects during the typesetting, hence they had to memorize it at the beginning of each attempt. This was motivated by the need to bring the subject closer to the natural way of typing, where one types spontaneously. The only difference with the regular typesetting mode was the instructions to the participant to refrain from using backspace button and ignore typos since we were interested in physiological events associated with error perception and not in those related to reaction. All sentences, had to be written using lower-case letters with a full stop at the end. Each session, which consisted of typing one sentence, was followed by a short-time break.

### Pragmatic Typing Protocol

Since it was necessary to compare the individuals’ typesetting performance with and without the EDS and regarding the time needed to correctly type a given text, we included an additional round of control experiments. During this round each participant had to “type” the same sentences, but this time using the gaze-based keyboard in its regular mode (without error-detection assistance) and with the instruction that each sentence would be considered complete only when it had been correctly typed. In this mode, participants were allowed to use the backspace button during the typesetting; all other parameters remained unaltered.

### Data Analysis

The concurrent data streams (after the necessary pre-filtering for the EEG traces) were segmented into epochs. Each epoch contained the physiological responses starting 200 ms before the onset of visual key pressed and lasting for 700 ms. Since the erroneous responses were much fewer than the correct ones the two categories were not equally represented. To alleviate this situation, we employed the SMOTE^17^ algorithm that creates synthetic patterns from the less populated category. For analysing the brain responses, the approach of Discriminant Spatial Patterns^18^ was adopted so as to improve the detectability of ErrPs. For analysing the gaze-related signals, the Hjorth descriptors^19^ were employed for characterizing the derived timeseries that incorporate the successive displacements of the gazing position. Two independent models based on Support Vector Machines (SVMs) with linear kernel^20^, were trained to discriminate between correct and erroneous typesetting. The one operated on feature vectors extracted from the single epochs of brain activity and the other on the feature vectors extracted from the associated epochs of gaze-related activity. The individual outputs were fused to realize the final error detection. Technical details are presented in the corresponding *Materials and Methods* sections.

### System Adjustment and Evaluation

Accuracy is typically employed in classification tasks to evaluate the performance of the system. However, in class imbalance situations (like it is the most probable scenario for our EDS), sensitivity/specificity pairs offer a more reliable evaluation of a given classification model. The Utility metric^21^ is a suitable composite measure, which in addition to specificity and sensitivity incorporates the dwell typing time modulated by the mistyping probability as well. It is widely used for describing the performance of BCI-spellers, and here it was used for tuning the EDS and providing the final unbiased justification of it’s gain via a Monte Carlo cross-validation scheme.

After the classification scheme was decided, on data obtained from the first typing task (i.e. using the error-aware keyboard), an error-aware simulation took place. During the simulation the time interval for typing a given sentence in error-aware mode was calculated and then compared against the time interval using the regular gaze-based keyboard. To avoid biases, in the former case, we first trained the incorporated EDS in a “Leave-One-Sentence-Out” (LOSO) manner (i.e. using the epochs from the rest 19 sentences) and, then, simulated “off-line” the operation of the error-aware keyboard on the sentence. Finally, taking into consideration both the false positives and negatives of the EDS system (and the associated gain/loss in time), we predicted the necessary time interval for our typesetting approach. The simulation and time calculation procedures are detailed in *Materials and Methods: Typing Time Estimation*.

## Results

### Physiological Findings

**Figure 2:**
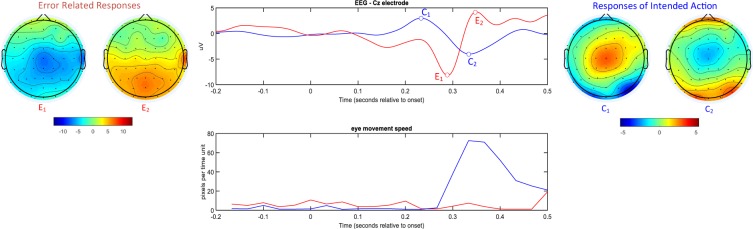
Single-subject averaged brain activation traces for the correct (blue) and wrong (red) selections of buttons are shown in the top middle panel. Particular latencies are indicated on these traces (E: error; C: correct) and the corresponding topographies have been included in the top left/right panels. The traces shown in the bottom middle panel reflect eye-movement activity (derived by averaging correspondingly across the epochs of gaze-related signal). Zero time indicates the instant that the typing of the current letter has been completed and the eyes are free to move towards the next letter.

Among the principal objectives of this study was to understand and characterize the physiological responses associated with the perception of an error during the gaze-based typing procedure. Figure 2 conceptualizes our main empirical findings about the neural correlates and the eye-motion patterns that served as the basis for deriving error-detection signatures from the recording data streams. Using single-subject data, the averaged responses for both brain activity and an eye movement descriptor are presented for the case of correct and erroneous typesetting. Regarding the brain activity patterns, it becomes apparent that the main components of the error-followed response is a negative deflection around 300 ms (with a frontocentral scalp distribution) followed by a positive peak 100 ms later (with a centro-parietal scalp distribution). These latencies have been defined with respect to stimulus onset, which is the time instant that the gazed letter is registered and its preview is shown to the user in the corresponding ribbon. The shown activation pattern is topographically in accordance with the relevant ErrP literature^22^, but differs slightly in the timing. In the case of correct typesetting, the brain activation shows a pattern that deviates from the anticipated null response. Specifically, a moderate biphasic response can be seen, consisting of a positive deflection around 200 ms and a negative one around 300 ms. Its topographical representation points to a cortical source located centrally. The explanation for the observed neural patterns is naturally suggested by the temporal patterning of the associated eye activity. As it can be seen (Fig. 2; bottom-most traces), the averaged profile of eye-movement speed is suggestive of apparent eye-motion only in the case of a correct typesetting, and specifically well after the gazed letter is typed. It becomes obvious that after a typing error subjects adjusts slightly their gaze, since their intention has been marginally misinterpreted, while in the opposite case they have to type the next letter which is most probably located, on the screen, far from the previously gazed position. Nevertheless, the successive typesetting of nearby letters is a possible scenario as well (e.g. in the case of word “was”). Finally, it should be underlined that the scalp topographies (included in right most panels) after the correct typesetting comply with cortical generators lying in a brain region that is known to causally relate with the eye-movements^23^. More importantly, they cannot be attributed to ocular artefacts generated by the eye movement.

**Figure 3:**
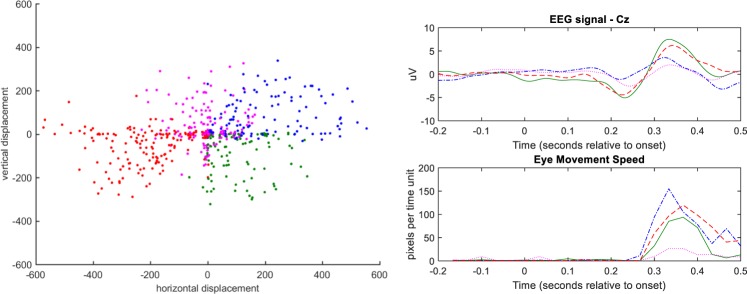
(Left) Scatter-plot of gaze centre displacements (derived by integrating the derivatives of eye position coordinates within a time interval that includes the key registration at 0 latency). Each dot indicates the main direction of the eye after a correct typesetting of a single letter. The point swarm has been partitioned into 4 groups, and the membership of each dot is indicated by colour. The associated brain-signal and eye-movement activity traces have been grouped accordingly and their (sub)averages are indicated in top right and bottom right respectively using the colour code defined in scatter-plot.

To further investigate the association between the EEG activity and gaze shifts, we adopted a data analytic procedure, in which the variability in eye-movement directionality was first coarse grained and then utilized to condition the grouping of brain activity traces. Figure 3 includes the results from analysing the single epoch data of the same participant as in Fig. 2. Using the aggregated gaze displacement, from −200 ms to 500 ms around each correct letter registration, we applied k means algorithm to group the eye movements into k = 4 distinct directions (Fig. 3; most left). The derived grouping was then applied to the eye movement speed profiles and, also, to the corresponding EEG traces. Finally, the four distinct prototypical traces, for both types of physiological activations were presented in a contrasting manner (Fig. 3; right bottom and top). It becomes evident that EEG-traces do not show any polarity inversion with the change of eye-movement direction (blue/red waveform corresponds to right/left) that would have been the case if contaminated by ocular activity artefacts. On the contrary, the temporal patterning at Cz sensor seems to follow the profile of eye-movement speed.

### SVM classification for predicting typesetting errors from physiological activity

Having justified the potential of brain activity and gaze-movement patterns for distinguishing between correct and mistyped letters, we proceeded towards implementing the idea of error detection by way of a machine learning algorithm. Features from the EEG-related epochs or/and the concurrent gaze-based traces were used in the context of an SVM-classification scheme. Depending on the type of the features and the way they were combined, four different classification schemes were realized and validated using the standard performance metrics of sensitivity and specificity. In the first scheme only EEG features were considered, while in the second only eye movement descriptors were employed. In the other two schemes, denoted as “early” and “late” fusion, the error detection was based on both data streams. The corresponding features were treated jointly by a single SVM classifier in the former case, while in the latter case they were fed separately into two distinct SVM classifiers where the most dominant confidence score indicated the classification result. As Table 1 indicates, the classification using brain activity pattern tends to emphasize specificity, while eye movement descriptions emphasize sensitivity.

**Table 1:** Performance metrics for the classification task of discriminating between correct and erroneous typesetting based on EEG traces and gaze-movement patterns (used both separately and jointly).

Subject ID	EEG		Eye Motion		Early Fusion		Late Fusion	
	Sensitivity	Specificity	Sensitivity	Specificity	Sensitivity	Specificity	Sensitivity	Specificity
S01	73.41	83.19	97.82	51.39	78.95	85.96	91.51	78.00
S02	75.54	87.97	90.14	71.94	80.48	89.34	85.55	87.00
S03	85.52	93.20	89.36	82.83	87.40	94.00	89.50	93.13
S04	74.76	85.18	87.45	70.26	80.13	85.18	83.84	81.92
S05	76.38	85.90	88.34	75.71	82.58	89.83	85.25	89.96
S06	76.69	80.09	95.68	63.95	88.92	75.82	92.65	73.69
S07	61.61	78.79	83.98	67.89	71.71	77.79	79.37	75.57
S08	69.31	83.76	94.21	71.35	89.87	85.30	90.26	83.76
S09	67.39	82.36	87.73	77.18	78.64	83.94	82.71	83.70
S10	70.47	81.41	80.77	80.56	79.35	80.80	79.76	82.69
**Average**	**73.10**	**84.18**	**89.54**	**71.30**	**81.80**	**84.79**	**86.04**	**82.94**

Tabulated are the results averaged from 100 repetitions of Monte-Carlo cross validation. Four implementation scenarios have been validated, for each subject independently.

### Incorporating the SVM classifier(s) in the gaze-based keyboard

**Figure 4:**
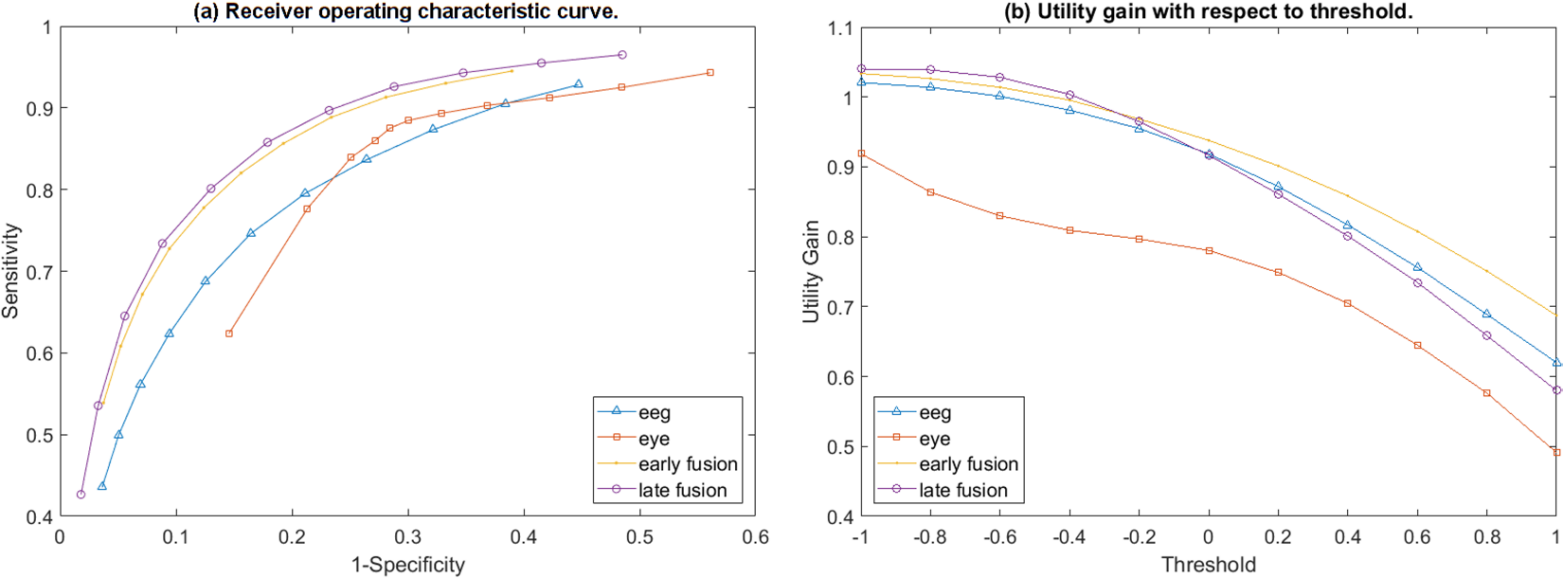
The grand average sensitivity and specificity values (**a**) along with the Utility gain (**b**), after 100 Monte-Carlo cross validation repetitions, with respect to threshold moving within the normalized SVM margins.

The detection of error responses is the key procedure in the realization of an error-aware gaze-based keyboard. Therefore, the classification accuracy of the incorporated SVM-algorithm, alone, was not enough for the full justification of the developed error detection system. This is because typesetting errors occur at low probability (roughly in 1 out of 10 characters) and hence the SVM-model has to deal with an imbalanced classification task. The Utility metric offers a meaningful way, to weight appropriately both the sensitivity and specificity and, simultaneously, to incorporate the cost of typing time and the error chance. This metric served as a (inverse) cost function to optimize the functionality of the linear SVM, by adjusting the position of the separating hyperplane. As a result, the classifier incorporated in the error-aware keyboard did not perform optimally regarding the accuracy in error detection task, but led to higher Utility gain (the ratio Utility_EDS_/Utility_regular_). Figure 4a includes the standard ROC curves for the four classification schemes, using as threshold a value within [−1, 1] for classifying an instance as erroneous or correct. It is evident that the late fusion scheme shows always superior performance regarding specificity and sensitivity. Figure 4b shows the associated Utility gain as a function of the threshold. A constantly increasing gain, is associated with lowering the threshold in all four schemes. This trend in gain is followed by an increase in specificity, which is apparently more important than the sensitivity in error detection. As expected, lowering the threshold beyond −1 results into a gain decrement. In summary, when the two SVMs in the late fusion scheme, uses the most negative threshold value, that is −1, the best performance of the error-aware gaze-based keyboard will be achieved.

A more detailed picture is provided in Table 2 where the results obtained at the optimal threshold by means of Utility gain, for all participants, have been included. The following empirical facts needs to be underlined. EDS based on features from the EEG, but not from the eye-activity, may lead to enhanced user’s experience in terms of time (utility gain higher than one). The late fusion EDS scheme systematically provides the best results, which on average correspond to a 4% increase in Utility gain. Finally, all accuracies are well above the random chance (which is defined by the typing error chance).

**Table 2:** Columns 2–6: chance that the eye-tracker will interpret user’s intention falsely and utility gain for the four classification schemes.

Subject ID	Typing Error Chance	Utility Gain				specificity	sensitivity	accuracy
		EEG	eye motion	early fusion	late fusion			
S01	11.60%	1.02	0.69	1.05	1.05	98.64	45.60	92.48
S02	7.92%	1.01	0.93	1.02	1.03	98.55	45.40	94.33
S03	6.36%	1.04	0.99	1.04	1.04	99.28	71.00	97.48
S04	11.60%	1.04	0.93	1.04	1.05	98.53	46.50	92.48
S05	8.87%	1.02	0.91	1.04	1.04	98.94	50.60	94.66
S06	13.67%	1.03	0.92	1.03	1.03	96.68	39.20	88.81
S07	13.50%	0.99	0.92	0.99	1.02	97.97	24.10	87.99
S08	9.24%	1.00	0.94	1.03	1.03	98.89	28.70	92.39
S09	6.16%	0.98	0.91	0.99	1.01	97.66	35.40	93.82
S10	13.84%	1.03	1.01	1.05	1.05	98.84	31.20	89.44
**Average**	**10.27%**	**1.02**	**0.92**	**1.03**	**1.04**	**98.40**	**41.77**	**92.38**

Columns 7–9: classification performance metrics at the optimal threshold for the late fusion classification scheme. Tabulated are the results averaged from 100 repetitions of Monte-Carlo cross validation.

### Simulated implementation of the EDS

In an attempt to compare the time efficiency of the error-aware gaze-based keyboard against the regular one, we contrasted the time that it would be needed for users to type a given sentence with our EDS system erasing the erroneously typed letters and them re-typing the letters immediately (T1 task), with the time needed for them to use backspace to correct the errors (T2 task). Table 3 presents the average (AVG) necessary times regarding the regular gaze-based keyboard, for each participant separately, and the corresponding difference with the error-aware typesetting. It becomes apparent that, on average, users require 2.7 s less to type a sentence which would require 29.36 s approximately. This leads to a 9.3% increase in typing speed (which is statistically significant with a p-value = 0.032 based on Wilcoxon signed rank test), that remarkably differs from the theoretical gain of 4% that was calculated through the Utility metric. The reason for this deviation lies on the fact that the utility metric assumes identical key press times for letter and backspace buttons, which is not the case according to our empirical results (exact times are included in Supplementary Table 2). In addition, of interest is the correlation between the obtained time gain and the user’s probability of making a typo. Actually, the gain from the EDS is upper bounded by the misinterpretation probability of the eye-tracker^24^. In other words, users prone to errors during gaze-based typesetting tend to benefit more from our hybrid BCI system.

**Table 3:** Chance that the eye-tracker will interpret user’s intention falsely in the two typesetting procedures (T1 and T2), the average gain in time (accompanied by the respective percentage) that is obtained by the EDS system taking advantage of the late fusion classifier and the average time required to type one sentence in both tasks.

Subject ID	Typing Error Chance		Gain (T2-T1)		Average Sentence Time	
	T1	T2	Time (seconds)	Percentage	T1 (seconds)	T2 (seconds)
S01	11.60%	6.48%	1.23	4.51%	26.07	27.30
S02	7.92%	4.14%	4.17	13.99%	25.64	29.81
S03	6.36%	8.74%	7.33	22.96%	24.60	31.93
S04	11.60%	8.44%	7.53	21.68%	27.21	34.74
S05	8.87%	9.05%	4.92	16.12%	25.61	30.53
S06	13.67%	3.56%	−0.78	−2.90%	27.72	26.94
S07	13.50%	5.83%	0.44	1.52%	28.51	28.95
S08	9.24%	3.36%	−0.11	−0.42%	26.20	26.09
S09	6.16%	2.55%	−1.38	−5.41%	26.88	25.50
S10	13.84%	9.50%	3.74	11.74%	28.11	31.85
**Average**	**10.27%**	**6.17%**	**2.70**	**9.23%**	**26.67**	**29.36**

The results are obtained according to a Leave-one-Sentence-out cross validation manner.

## Discussion

Eye tracking systems have been greatly improved and, nowadays, offer a viable and affordable communication channel, especially for people lacking fine motor skills. However, input by eye-gaze is challenging due to several technical issues that may introduce ambiguity and compromise the accuracy in detecting the eye-position^25^. Therefore, an interaction-context specific optimization of the eye tracking procedure is considered necessary for achieving a satisfactory experience for the user. In this work, we adopt a different strategy in order to achieve a more natural incorporation of the eye-gaze input. We introduce a hybrid BCI system that takes advantage of humans’ inherent ability to perceive an erroneous action and their behavioral reaction for revising it, in a very intuitive and effortless way. The novel error-detection system pieces together the ErrP-response (that reflects the first cortical reaction to an error) with the eye-movement patterning (that reflects re-attempting to typeset the character), and may be readily adopted in gaze-based keyboards in order to offer higher typing rates.

Our experimental data showed that even though the interpretation of user’s eye movements may allow for detecting typos with relatively high accuracy, it is only the decoding of EEG patterns that can lead to sufficiently high detection rates so as to improve the user experience. More importantly, it is the hybridization of the two modalities that ultimately offers the most promising solution. Apparently, the suggested scheme may appear unconventional since in the majority of neuroscientific studies the participants are instructed to confine the ocular movements as much as possible, and whenever eye-activity is registered this is mostly done for EEG-denoising purposes. In our case, nevertheless, the eye movements are an integral part of the process underlying the typesetting and, hence, the eye-activity information complements the ErrPs-event detection.

A further justification of this finding should be attempted at this point. First of all, the eye movement related artefacts are not seen in the formed averaged responses (both for the correct and erroneous typesetting; refer to Fig. 2). In the case of erroneous typesetting, and according to the data stemming from the eye tracking, there is very limited eye movement activity associated with the perception of typos, that does not affect the brain response in the electrodes where ErrPs are typically met. On the contrary, in the case of a correct key registration, the eye movement is significant and could, in principle, affect the EEG responses. To rule this possibility out, we studied the brain responses with respect to the direction of the eye movement (Fig. 3). Having in mind that opposite eye movements (e.g. eye moving upwards versus downwards) produce electrical activity patterns with reversed polarity^26^ and such an effect would disappear after averaging, we confined averaging within the subset of trials that are related with eye-movements towards the same direction. By comparing the (sub)averaged brain responses from different eye-movement directions, we verified that the eye-related artefacts do not contaminate the brain-activity signal at the electrodes of interest. This does not mean, though, that oculographic artefacts are not present at recording sites over the pre-frontal cortex. Moreover, a spatial filter is designed to enhance the ErrP detection and diminish artefact-related effects, using data segments within the examined temporal window, that is [−200, 500] ms around the latency of a character registration. This filter, that conditions the data for the SVM, is activated only during the [0, 500] ms interval. Finally, we underline here that the signals from the two modalities were, also, used independently (within the same data-learning framework) for building an EDS and the performance was inferior in both cases (Table 2).

Having in mind to investigate the physiological events associated with the perception of an error during gaze-based typing, we implemented an experiment that ensured the elicitation of error-related potentials and their subsequent robust detection. The developed EDS may seem contradictory to the established typing mode, where every erroneous key press is typically followed by an immediate backspace press. A less common, but also quite effective, typing mode involves successive key presses, during which mistypes are ignored and collectively corrected at the end of each period. This mode, along with the optimal hybrid EDS, appears as the most promising typing paradigm that could be fully benefited from the introduced approach.

The proposed error detection system could be incorporated in any gaze-based keyboard. Although eye trackers provide a convenient and relatively reliable way for registering users’ gaze they are still extremely error-prone in the case of certain target groups, such as those who are suffering from eyelid ptosis or diplopia^27^. The proposed EDS seemingly benefits from high error chances, since our experimental data indicate an association between typing speed gain and typing error chance, as Table 3 shows. Consequently, it is expected to have a significant merit for the users who face difficulties in operating an eye-tracking device.

The main aim of this study was to introduce a novel Error Detection System for gaze-based keyboard that has the potential to improve the overall typing speed. We have shown that certain physiological events, associated with both brain and eye activity, can mark an unintended visual key press and therefore serve as the basis for an error correction system. It is important to note at this point, that the essence of our work lies in detecting users’ intentions. From this perspective, there is a major difference between the presented Error Detection System and an automatic Error Correction System (i.e. proofing) that may offer even higher typing speed. There will be, however, certain occasions that an automatic spell proofing tool will be less reliable since it enforces the users to write “correctly” by preventing them from using shortened language expressions and idiosyncratic writing style. Having said that, we need to mention, also, the possibility that, in a future implementation, such a spell proofing system could be activated occasionally by our error-aware gaze-based keyboard.

## Materials and Methods

This section includes additional details on all technical aspects of this work. Initially the experimental protocol and it’s reasoning are explained. Next, the methodological framework for processing the eye motion and EEG signals is introduced, followed by a detailed description of the classification procedure and its verification. Finally, the method for calculating the time required to write the sentences in both error-aware and regular keyboard settings is explained. This method served as the basis for the final justification of the introduced error-aware gaze-based keyboard.

### Experimental Protocol

This study includes experimental data from 10 subjects aged 20 to 45 years (mean 32 ± 4; 4 female). The participants joined the experiments voluntarily, had no known prior or current pathological neurological condition and their vision was normal or corrected to normal. All participants were familiar with physical keyboard devices and therefore aware of the qwerty keyboard layout.

Initially, the experimenter shortly described the experiment and its purpose to the participants. It was stressed to the participants that these experiments had been designed so as to explore their activity during erroneous actions and not to test their ability in typing tasks. For this reason, a challenging function-mode was used for the keyboard, in which the letters were being “locked” relatively fast (i.e. after detecting an eye fixation within the area of the letter for just 0.5 seconds) and not progressively as in the standard mode.

It was made clear to the participants that in case of an unintentionally typed letter, they should ignore the erroneous key press and retry typing the correct letter until the whole sentence is completed. This would lead to a sentence that contains, among typos, all the letters of the intended sentence in the correct order (i.e. the corrected version should be readily recovered by simply deleting the mistyped letters). The adopted strategy offered us as a way to ensure that the erroneous visual key presses had been perceived as such by the user. Finally, the subjects had been instructed to refrain from head and other type of body movements, as much as possible, in order to avoid artefacts (such as jaw clenches and hand/feet movements) that would distort the EEG signal.

Brain’s electrical activity was registered via the EBNeuro EEG device, which offers 64 wet electrodes placed according to the 10-10 international system (Supplementary Fig. 1). Gaze-related information was registered via the SMI myGaze eye-tracker. The sampling frequency was 256 Hz and 30 Hz for the EEG and eye-tracker device respectively. The Lab Streaming Layer^28^ software supported the synchronization of the streams with a sub-millisecond accuracy.

The experimental procedure was designed and performed in accordance with the relevant guidelines and regulations, particularly those set out in the Declaration of Helsinki pertaining to the ethical treatment of human subjects. Participants signed informed consents, and were instructed on their rights as participants, including the right to withdraw from the experiment at any time without fear of negative consequences. The study protocol has been approved by the Ethics Committee of the Centre for Research & Technology Hellas (REF NO: ETC.COM_28).

### Eye Motion Descriptors

Exploiting the successive eye-position coordinates, we calculated the length of the trajectory that eyes had traversed on the screen along time. The derived “accumulated distance” timeseries, constituted the basis for the feature extraction step regarding the gazing information. Hjorth parameters^19^, served as the final descriptors for the aforementioned timeseries. These descriptors are often employed to describe the dynamical behaviour of a signal by incorporating its statistical properties within the time domain. In general, for an initial signal x(t), the corresponding Hjorth descriptors are defined as follows: *activity*(*x*(*t*)) = *var*(*x*(*t*)), $mobility(x(t))=\sqrt{\frac{var(\frac{\partial x(t)}{\partial t})}{var(x(t))}}$ and $complexity(x(t))=\frac{mobility(\frac{\partial x(t)}{\partial t})}{mobility(x(t))}$. In our case, these three descriptors described not only the time course of successive displacements, but also encapsulated the speed and acceleration (first and second order derivatives of distance) in a sophisticated way. More specifically, the Activity parameter (that expresses total power of the signal^29^) was associated with the total length of the trajectory. The Mobility parameter (that is interpreted as an estimate of the signal’s mean frequency) was reflecting the alterations of the eye motion speed. Finally, the Complexity (that is known to reflect the bandwidth of the signal) was reflecting the motion irregularities.

### EEG Spatial Filtering

Event Related Potentials (ERPs) are among the most popular brain signatures for establishing brain-computer interaction. The wide range of ERP-BCIs extend from P300 spellers^30^ and Error-Related Potential based auto correction systems^31^ to fixation-related potentials for relevant target identification^32^. Actually, an ERP signal is a recorded brain response that is the direct after-effect of a specific event (e.g. the perception of an erroneous action). ERPs typically are of low Signal-to-Noise-Ratio (SNR) brain responses therefore an averaged response across a large number of trials is presented. The averaging procedure guarantees that the noise is eliminated and the produced signal is “practically” free of noise. There are two certain conditions that should hold so that the averaging produced noise-free signal. Firstly, the signal of interest should consist of phase-locked responses with invariable latency and shape. Secondly, the background noise should follow a random Gaussian process of zero mean, uncorrelated between different recordings and not time-locked to the stimulus^33^. We know that EEG measures the electric potentials on the scalp. The neural responses of interest are reflected in several electrodes placed over the scalp. Utilizing the spatial information and in an effort to increase the SNR of the ERPs we employ the spatial filtering approach. More specifically, we seek the linear combination that will maximize the Fisher’s separability criterion^18^. Assuming two groups of epochs, ErrPs and non ErrPs, denoted by **{X}** and **{Y}**, the optimal spatial filter can be obtained by solving the following optimization problem:

1 $${{\bf{w}}}^{\ast }=\mathop{{\rm{a}}{\rm{r}}{\rm{g}}{\rm{m}}{\rm{i}}{\rm{n}}}\limits_{{\bf{w}}}\frac{{\bf{w}}{\boldsymbol{(}}\bar{{\bf{X}}}-\bar{{\bf{Y}}}{\boldsymbol{)}}{\boldsymbol{(}}\bar{{\bf{X}}}-\bar{{\bf{Y}}}{{\boldsymbol{)}}}^{{\rm{T}}}{{\bf{w}}}^{{\rm{T}}}}{{\bf{w}}({{\bf{C}}}_{{\bf{X}}}{\boldsymbol{+}}{{\bf{C}}}_{{\bf{Y}}}{\boldsymbol{)}}{{\bf{w}}}^{{\rm{T}}}}$$

where $\bar{{\bf{X}}}$ and $\bar{{\bf{Y}}}$ denote the average responses and **C**_**X**_ and **C**_**Y**_ the average noise level for the groups **X** and **Y** respectively.

### Preprocessing and Epoching

It is known that ErrPs are embedded into low frequency components^34^, so zero-phase bandpass filtering within (1–16)Hz range was applied to the EEG recordings. Then the recordings, both from EEG and eye-tracker devices, were segmented into epochs that were aligned with the visual key presses. Each epoch (single trial response) contained the segments of brain activity and eye-position trace corresponding to the time window [−0.2 0.5] seconds around a visual key press. In order to assign the proper label (correct or erroneous) to each epoch we compared the pressed letter with the one that should be pressed in order for the sentence to be typed correctly having in mind that previous errors should be ignored. For instance, consider that the sentence the subject should type was “my dog is brown” and the participant wrote “muyb dog ias browqn” instead. In this case the epochs corresponding to the letters u, b, a and q would be labelled as erroneous, while the rest ones as correct.

### Coping with Class Imbalance

Since the number of erroneous visual key presses is much lower than the number of correct ones we had to deal with a classification problem of imbalanced classes. This could lead to a classifier that classifies all observations as correct (i.e. the majority class). To tackle this, we followed the opposite tactic. From the minority class we generate new observations, referred to as minority oversampling, until the number of observations in both classes was equal. To perform this approach we employed the well-known Synthetic Minority Oversampling Technique (SMOTE)^17^. We must note that the SMOTE algorithm was performed on the band-pass filtered EEG data, in case of the brain activity. Spatial filtering is a data driven procedure that takes labels into account and therefore benefits form the oversampling. Similarly, in the case of eye-motion epochs, the SMOTE algorithm operated on the associated distance timeseries.

### Classification

Each single epoch was associated with two feature vectors; one from the eye-tracker and one from the EEG scanner. In the latter case, the feature vector corresponded to the temporal pattern that was mined by means of spatial EEG filtering. This step was accomplished by synthesizing a “virtual sensor” that would reflect the ErrP response with SNR higher than the individual sensors. The first discriminant component was extracted via eigen-analysis of the problem formulated in eq. (1) and utilized as the spatial filter. For the eye-motion signals, each epoch was described by means of Hjorth descriptors acting on the trajectory of the on-screen displacements. The choice of Hjorth descriptors was motivated by the assumption that when users managed to type a letter correctly their gaze would shift fast towards the next desired letter, while in the opposite case, they would slightly adjust theirgaze in order to type the intended letter. Initially the data, were split into train and test sets. The train set was used in order to create the augmented train set with synthetic data. Then, the augmented train set was used in order to calculate the Discriminant Spatial Patterns (DSP) weights and Hjorth descriptors. Thereafter, three separate SVMs were trained that operated on normalized (zero mean; unit variance) features. Two regarding each modality and a third one for the concatenation of features (“early fusion”)^35^. An additional “late fusion” scheme was also realized, that combined the output of the two uni-modal SVMs in order to form the final classification decision. The test set consists of ten erroneous responses and a specific amount of correct responses so as to maintain the initial ratio of correct over incorrect visual key presses. Both normalization and DSP parameters where elusively mined from the train set and then applied on the test set. In order to evaluate the classifier performance, a monte-carlo cross validation approach was performed where the splitting and testing procedures were repeated 100 times.

### Typing Time Estimation

It was straight forward to calculate the required time that each participant needed to type each sentence using the regular keyboard (T2). Initially the average time needed in order to press the backspace key was calculated empirically by T2 recordings and is denoted as *b*_*avg*_. Then the edit distance between the typed and the intended sentence was calculated, denoted by *d*. Typically, the edit distance was 0 since the indications to subjects stated that the sentence should be typed correctly (or perceived as such). However, there were certain occasions that subjects ignored the erroneous key press. Then the time difference between the first and the last key press, *t*_2_, is calculated. Finally, the total time for T2 task is computed as *t*_2 _ + *d b*_*avg*_.

In case of the first task (error-aware keyboard), time difference between the first and the last key press, denoted by *t*_1_, is calculated based on the times derived during the recording procedure. Then through the simulation each key press is assigned with a label denoting whether it was classified as a correct or an erroneous. There are two cases that are of interest. In the first, a correctly typed letter is classified as wrong and therefore the users should retype it. These cases should be penalized by the average time required to press a letter button, denoted by *l*_*avg*_. The second case concerns erroneous key presses that are classified as such and therefore should not be penalized since the error-aware keyboard would automatically erase them. Finally, the total time for T1 task is calculated as *t*_1_ + (*d* − *k*_1_)*b*_*avg*_ + *k*_2_ *l*_*avg*_, where *k*_1_ corresponds to the number of times that the classifier indicates an erroneous response is successfully detected, *k*_2_ corresponds to the number of times that a correct response was indicated as an erroneous and *d* denotes the edit distance between the typed and the intended sentence.

## Electronic supplementary material

Supplementary Information

## Data Availability

The physiological data used in the current study are available at figshare, 10.6084/m9.figshare.5938714.v1.
