# Supplementary material for: An error-aware gaze-based keyboard by means of a hybrid BCI system
Source: Sci Rep. 2018 Sep 4;8:13176. doi: 10.1038/s41598-018-31425-2 (PMC6123473; doi:10.1038/s41598-018-31425-2)
Supplement: Supplementary file 1 — Supplementary Information [file 41598_2018_31425_MOESM1_ESM.docx]

**An error-aware gaze-based keyboard by means of a hybrid BCI system**

**Fotis P. Kalaganis**^1,2,*^**, Elisavet Chatzilari**^2^**, Spiros Nikolopoulos**^2^**, Ioannis Kompatsiaris**^2^**, and Nikos A. Laskaris**^1,3^

^1^Aristotle University of Thessaloniki, Department of Informatics, AIIA lab, Thessaloniki, 54124, Greece

^2^Centre for Research and Technology Hellas, Information Technologies Institute, MKlab, Thessaloniki, 57001, Greece

^3^Aristotle University of Thessaloniki, Neuroinformatics Group, Thessaloniki, 54124, Greece

[*kalaganis@csd.auth.gr](mailto:*kalaganis@csd.auth.gr)

# **TABLES**

**Supplementary Table 1.** The sentences that were provided to the participants for both typing tasks using both regular and error-aware keyboard.

| **ID** | **Indicated Sentence** |
| --- | --- |
| 1 | my favorite hat is blue. |
| 2 | their dog is brown. |
| 3 | the summer is hot. |
| 4 | the bride looked beautiful. |
| 5 | i can swim well. |
| 6 | flowers smell good. |
| 7 | the rat is in the tub. |
| 8 | i see a big snowman. |
| 9 | roses are red and violets are blue. |
| 10 | my bike is red. |
| 11 | the fox can jump. |
| 11 | the fox can jump. |
| 12 | earth has one moon. |
| 13 | the lion is up in the tree. |
| 14 | zebras like to eat grass. |
| 15 | seven is an odd number. |
| 16 | big bugs are scary. |
| 17 | i wish I had a million dollars. |
| 18 | nature is a good journal. |
| 19 | i have a yellow watch. |
| 20 | this will never end. |

**Supplementary Table 2.** The time required for a letter press in both T1 and T2 tasks accompanied with the average backspace time in seconds. We note that the average time for pressing backspace is higher than the average time for presssing a letter in both T1 and T2.

|  | **Average letter T1 (seconds)** | **Average letter T2 (seconds)** | **Average Backspace T2 (seconds)** |
| --- | --- | --- | --- |
| SubID:1 | 1,1474 | 1,1015 | 1,2876 |
| SubID:2 | 1,1426 | 1,2701 | 1,6022 |
| SubID:3 | 1,1175 | 1,1702 | 1,3307 |
| SubID:4 | 1,1703 | 1,3226 | 1,617 |
| SubID:5 | 1,1063 | 1,1422 | 1,4333 |
| SubID:6 | 1,1649 | 1,1604 | 1,4072 |
| SubID:7 | 1,1725 | 1,1814 | 1,4501 |
| SubID:8 | 1,1421 | 1,1368 | 1,3499 |
| SubID:9 | 1,1874 | 1,1113 | 1,6417 |
| SubID:10 | 1,1519 | 1,1744 | 1,496 |
| **AVERAGE** | **1,150** | **1,177** | **1,462** |

# **FIGURES**

**
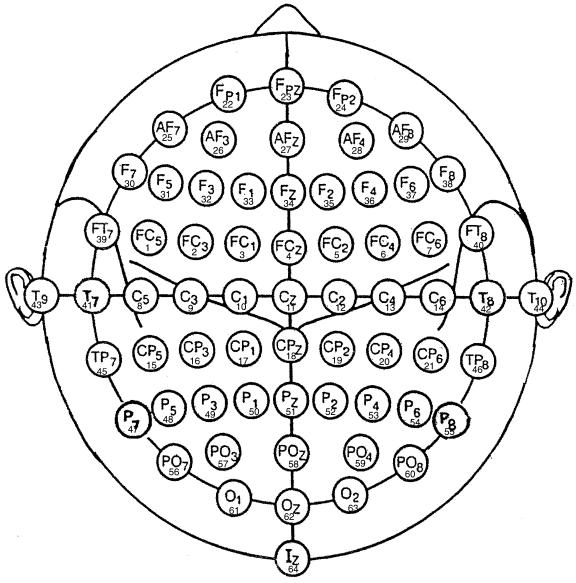
**

**Supplementary Figure 1.** Spatial location of electrodes for the EBNeuro headcap.

**
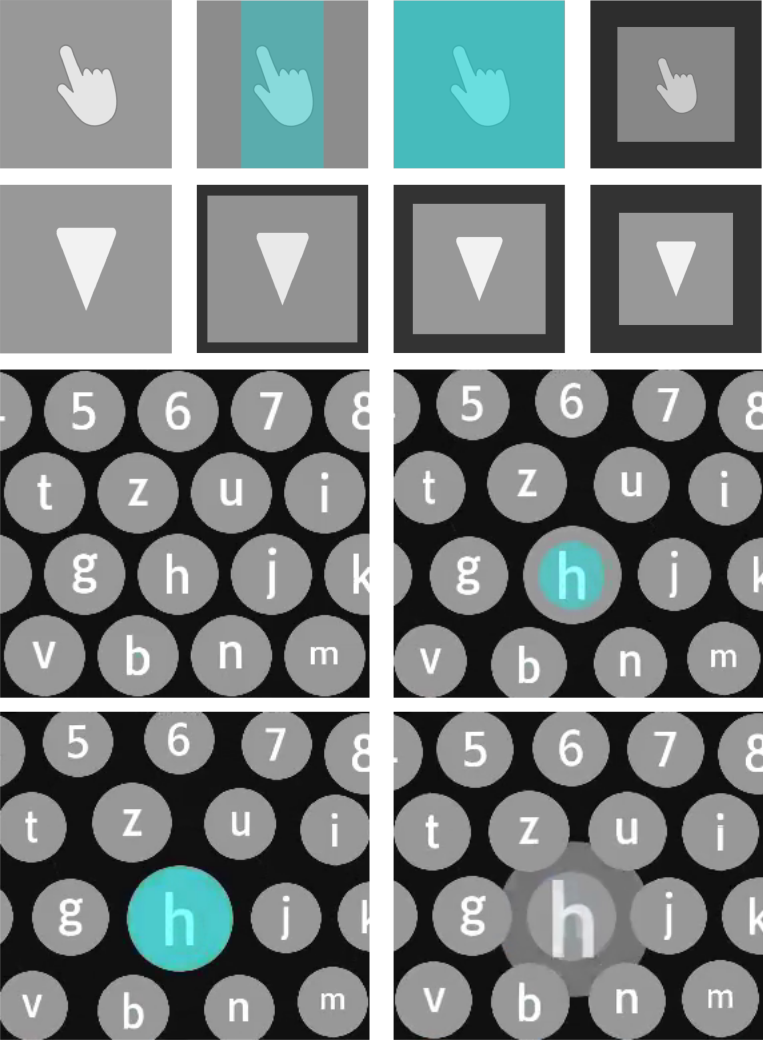

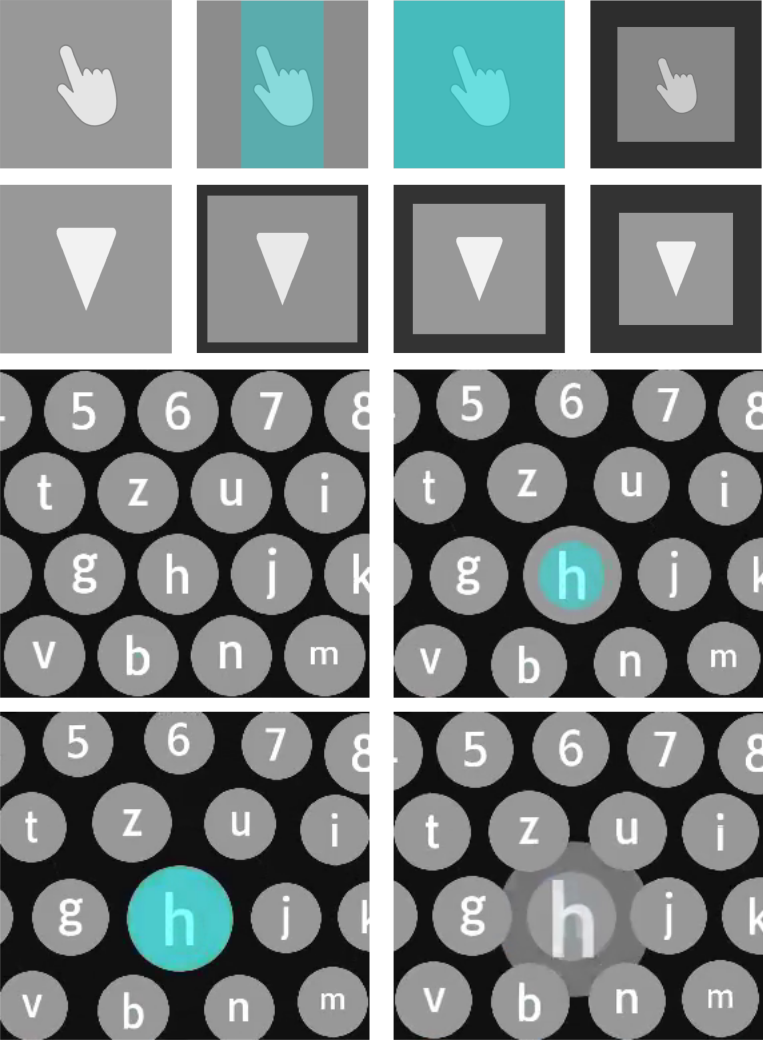
**

**Supplementary Figure 2.** (Left) The utilized gaze-based layout. (Center) The visual indication, which was deactivated in our experiments, that is typically employed in gaze-based keyboards to point out the key the user is gazing. It grows bigger and ultimately fills the gazed letter at the time it is registered. (Right) The visual indication showing that the corresponding key is registered.


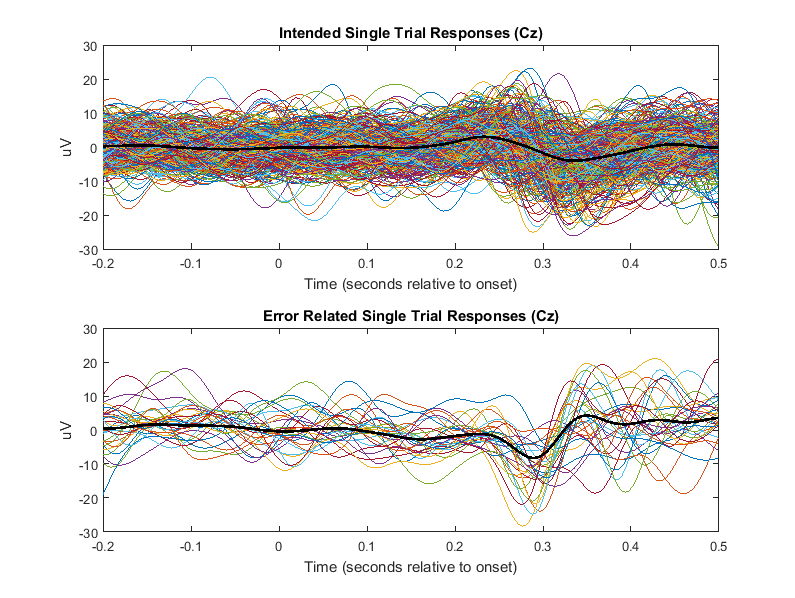


**Supplementary Figure 3.** Band-pass filtered (within [1-16] Hz ) single trial responses of intended (Top) and error-related (Bottom) actions. The bold black line in each case corresponds to the average waveform.
